# Development of Anterior Cranial Fossa Dural Arteriovenous Fistula and Supraclinoid Internal Carotid Artery Pseudoaneurysm Following Frontobasal Trauma: A Case Report and Literature Review

**DOI:** 10.7759/cureus.42330

**Published:** 2023-07-23

**Authors:** James Bai, Alex Kessler, Daniel Kawakyu-O’Connor

**Affiliations:** 1 Imaging Science, University of Rochester Medical Center, Rochester, USA; 2 Radiology, University of Rochester Medical Center, Rochester, USA

**Keywords:** skull base trauma, anterior cranial fossa dural-avf, skull base fracture complications, traumatic pseudoaneurysm, cerebrovascular injury, frontobasal trauma, ica pseudoaneurysm, traumatic dural arteriovenous fistula

## Abstract

Cerebrovascular injuries resulting from frontobasal head trauma represent a range of imaging and clinical presentations. Severe cerebrovascular injuries such as vessel transection commonly present with profound neurological deficits and are often easily identified with routine imaging. However, small intimal injuries and dissections may be challenging to detect and may be clinically silent or masked by additional injuries in the setting of polytrauma. The onset of symptoms and complications from cerebrovascular injuries may be delayed from the time of initial presentation, and failure to recognize and diagnose these injuries may result in devastating outcomes if management is delayed. In this case report, we present a case of frontobasal craniofacial trauma that resulted in an anterior cranial fossa dural arteriovenous fistula (ACF-dAVF) and supraclinioid segment internal carotid artery (ICA) pseudoaneurysm.

## Introduction

Vascular injuries have been identified in 8.5% of skull base fractures in some case series, particularly when the sella turcica-sphenoid sinus complex and the petrous carotid canal are involved [1,2]. Resulting patterns of vascular injury include laceration or transection with hemorrhage, intramural hematoma, dissection, pseudoaneurysm, or arterio-venous fistula. Variable onset and presentation of these vascular complications often make them challenging to identify clinically, and they may result in significant and lasting morbidity and mortality.

Dural arteriovenous fistulas (dAVF) are uncommon vascular lesions with abnormal connections between arteries and leptomeningeal veins or a dural venous sinus [3]. They may be idiopathic or acquired from venous obstruction secondary to prior trauma, surgery, or dural sinus thrombosis. Most frequently, dAVFs occur at the transverse/sigmoid sinus or the cavernous sinus. Anterior cranial fossa (ACF)-dAVF are particularly rare (1-1.5% of intracranial vascular malformations and 5.8% of all dAVFs) and known to carry a high risk of intracranial hemorrhage (62-91%) due to venous drainage through fragile cortical pial veins [3-6]. To our knowledge, this is the first case report of a patient presenting with ACF-dAVF and traumatic pseudoaneurysm following skull base trauma within one month of the initial encounter. 

Traumatic intracranial aneurysms represent fewer than 1% of all intracranial aneurysms [7]. Although trauma may result in true aneurysms with all three arterial wall layers remaining intact, post-traumatic pseudoaneurysms present with only a thrombus occluding the partial-thickness defect at the site of the arterial wall injury. It has been reported that the rupture risk of post-traumatic intracranial aneurysms is 50% [8]. The mortality of surgical versus conservative management has been reported as 18% versus 41%, respectively [9]. The cavernous and supraclinoid internal carotid arteries (ICAs) are particularly vulnerable to pseudoaneurysm formation in frontobasal trauma [10]. 

## Case presentation

**Figure 1:**
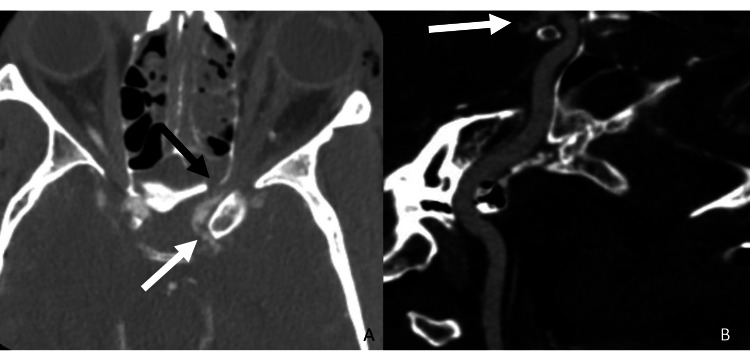
Supraclinoid ICA traumatic vessel injury Axial CTA neck (A) and curve reformat (B) demonstrate vessel irregularity and stenosis along the left supraclinoid ICA (white arrow) adjacent to the sphenoid sinus wall fracture (black arrow). ICA: internal carotid artery; CTA: computed tomography angiography

**Figure 2:**
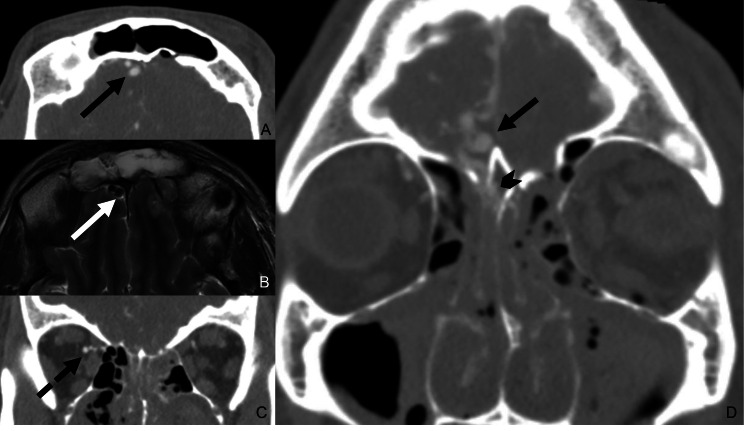
ACF-dAVF on CTA and MRI Axial (A) and coronal (C and D) CTA images demonstrates enlarged cortical veins along the right anterior cranial fossa (solid black arrow) and prominent right ophthalmic and ethmoidal arteries (dotted black arrow). The fistula point is centered at the fractured cribriform plate (black arrowhead). Axial T2 MRI sequence (B)  shows flow void of the enlarged cortical veins. ACF: anterior cranial fossa; dAVF: dural arteriovenous fistulas; CTA: computed tomography angiography

**Figure 3:**
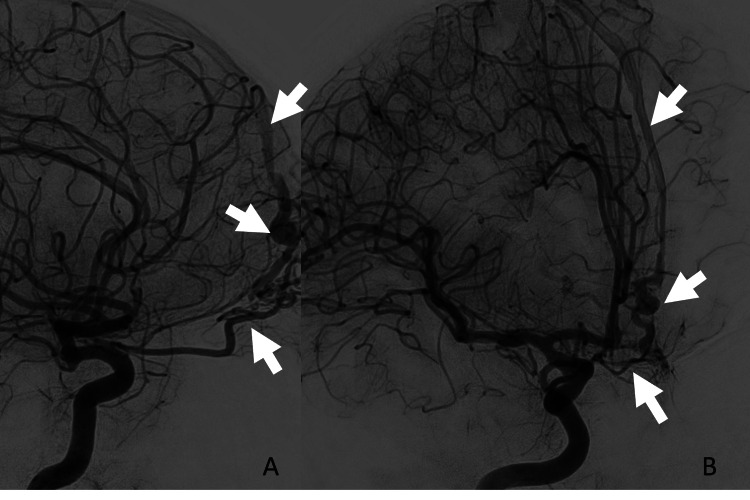
ACF-dAVF on DSA Lateral (A) and oblique (B) internal carotid artery injections on the initial DSA demonstrate a fistulous connection between the ethmoidal branches of the ophthalmic artery with early venous drainage into the superior sagittal sinus. ACF: anterior cranial fossa; dAVF: dural arteriovenous fistulas; DSA: digital subtraction angiogram

**Figure 4:**
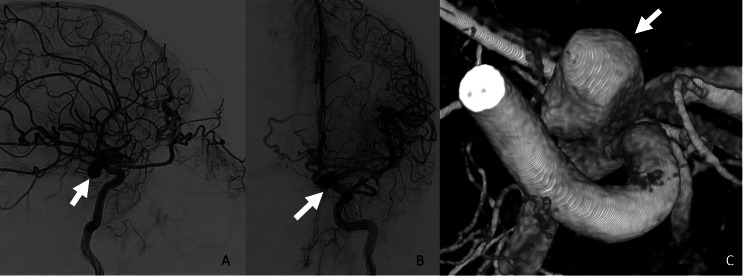
ICA pseudoaneurysm On the following DSA, Internal carotid artery injections in the lateral (A) and frontal (B) projections demonstrate interval development of a medially projecting pseudoaneurysm (white arrows), which is also shown on the 3D reconstruction from digital subtraction angiogram (C). DSA: digital subtraction angiogram; ICA: internal carotid artery

A 45-year-old male presented with extensive frontobasal and craniofacial injury after an all-terrain vehicle (ATV) rollover. Initial trauma computed tomography (CT) scans depicted bilateral naso-orbito-ethmoid complex fractures with fracture planes extending to the bilateral sphenoid sinus walls. Multi-compartmental intracranial hemorrhage, including subarachnoid hemorrhage and hemorrhagic contusions with basifrontal predominance, were also present. Subsequent CT angiography (CTA) of the neck revealed vessel wall irregularities and non-flow limiting stenosis involving the left supraclinoid ICA, and prominent cortical veins along the right ACF (Figures 1, 2). The following digital subtraction angiogram (DSA) performed three days later demonstrated arteriovenous shunting from the right ophthalmic artery to an enlarged cortical vein via ethmoidal branches that ultimately drains into the superior sagittal sinus consistent with a Borden III/Cognard IV ACF-dAVF (Figure 3). The same DSA exam also revealed non-flow limiting stenosis of the left supraclinoid ICA. A follow-up DSA performed two weeks after the initial encounter indicated interval development of a 9.1 mm x 6.5 mm medially projecting left internal carotid artery sidewall saccular aneurysm (Figure 4). The patient subsequently underwent balloon-assisted coil embolization of the left ICA pseudoaneurysm, followed by bifrontal craniotomy for disconnection of the ACF-dAVF a week later. Postoperative DSA demonstrated technical success with a resolution of arterial-venous shunting of ethmoidal arteries into the superior sagittal sinus and no residual filling of the treated ICA pseudoaneurysm. 

## Discussion

Cerebrovascular injury from blunt trauma can be subtle and difficult to detect at initial presentation. It can be stratified using a five-point scale Biffl cerebrovascular arterial injury grading scale based on the severity of vessel injury from mild intimal injury (Grade I) to vessel transection (Grade V) (Table 1) [11]. Grade I injuries spontaneously resolve in two-thirds of cases, whereas Grade V injuries are fatal [11]. Sequelae of post-traumatic vessel injuries such as dAVF and pseudoaneurysm typically present days to months after the initial encounter, and are often clinically silent and radiologically occult on the initial CTA or magnetic resonance angiogram (MRA) exams. There is no consensus in the literature on the timeline for a follow-up angiographic exam following normal initial CTA or MRA; even the mechanism and pattern of injuries would suggest a high pre-test probability for traumatic vessel wall injury. Some authors have suggested angiographic exams be performed one or two weeks after suspected vascular injury to limit the opportunity for false-negative results from initial imaging and the potentially life-threatening consequences of a missed vascular injury [10,12]. Other authors have suggested 6-12 months post-operative radiological follow-up for suspected iatrogenic vessel injury [13]. 

**Table 1:** Blunt cerebrovascular arterial injury grading scale

Vessel Injury Grade	Angiographic characteristics
Grade I	Mild intimal injury or irregular intima with luminal narrowing less than 25%
Grade II	Dissection with raised intimal flap/intramural hematoma/intraluminal thrombosis with luminal narrowing greater than 25%
Grade III	Pseudoaneurysm formation
Grade IV	Occlusive vessel thrombosis
Grade V	Vessel transection

In this case, the patient developed an ACF-dAVF within three days of the initial encounter, which is to our knowledge the first case report of a patient presenting with this condition within one month of the initial injury. In ACF-dAVFs, the cribriform plate is commonly the fistula point, in which afferent arterial supply arises from the distal ophthalmic artery and both anterior and posterior ethmoidal arteries, and venous drainage occurs through frontal cortical veins into the superior frontal sinus or posteriorly into the cavernous sinus or basal vein of Rosenthal [14,15]. In the absence of a more robust dural sinus in the ACF, the fragile pial cortical veinous drainage predisposes ACF-dAVFs for rupture and intracranial hemorrhage. ACF-dAVFs often present with associated enlarged cortical veins along the olfactory fossa. In our case, a prominent ophthalmic artery and fistula formation at the fractured cribriform plate are also seen (Figure 2). Varices of adjacent cortical veins may also be present in some cases. MRI may demonstrate flow voids of enlarged cortical veins in the ACF as evidence of dAVF [5]. DSA is the gold standard for diagnosing dAVF and typically identifies the presence of arterial-venous shunting from ethmoidal branches of the ophthalmic artery to enlarged cortical veins. The Cognard and Borden classification systems are the most commonly used to describe dAVF and are often used simultaneously. The Borden classification is based on the presence of (a) dural venous sinus/meningeal drainage, and (b) cortical venous drainage (CVD). The Congard classification is based on the description of (a) the direction of dural sinus drainage, (b) the presence of CVD, and (c) the type of venous outflow. In general, lesions are considered “aggressive” when CVD is involved [16], with the hemorrhage rate for these aggressive fistulas reported as high as 10% per year [16,17]. Treatment of high-grade lesions is typically performed through transarterial, transvenous, or combined endovascular techniques [18]. Surgical resection is generally reserved for difficult-to-access sites, such as the floor of the ACF [6,19]. Conservative treatment with surveillance imaging is often employed in low-grade lesions, and for lesions that are not amenable to endovascular or surgical therapy. 

Traumatic intracranial aneurysms may be true or false aneurysms. True aneurysms have preserved adventitia and partially disrupted and damaged intima, internal elastic lamina, and media, whereas false aneurysms/pseudoaneurysms result from disruption of all arterial wall layers and are contained by adjacent hematoma and perivascular connective tissues [20]. In particular, intracranial pseudoaneurysm has a high risk for rupture and is associated with a high mortality rate, and timely diagnosis can prevent devastating outcomes. Intracranial pseudoaneurysms have variable clinical presentations, including epistaxis, headache, seizure, unilateral blindness, and other neurological deficits [10]. Epistaxis is a common symptom in cavernous ICA pseudoaneurysm due to its proximity to the sphenoid sinus. Intracranial pseudoaneurysm may result in cranial nerve II, III, IV, V1, V2, and VI palsies due to proximity to the cavernous structures [10]. As with dAVFs, DSA is the gold standard for diagnosing intracranial pseudoaneurysms, as CTA and MRA have limited sensitivity for detecting small aneurysms and pseudoaneurysms [10]. If the initial imaging is negative, angiography may be repeated because pseudoaneurysms may develop days to weeks after the initial injury, although a specific timeframe for follow-up has not been established. When identified, pseudoaneurysms are typically treated with endovascular technique through the use of coil embolization, stent-assisted coil embolization, flow-diversion, or parent artery occlusion. If endovascular treatment is unsuccessful or the pseudoaneurysm is in a location that cannot be accessed easily, surgical intervention is employed through the use of clipping, wrapping, bypass, or ligation of the parent artery.

## Conclusions

This report is a description of a rare case of ACF-dAVF and ICA pseudoaneurysm that developed in the days following significant frontobasal craniofacial trauma as an opportunity to discuss the clinical presentation and associated imaging findings of these lesions, and to provide a better understanding of the pathogenesis and management of these and related post-traumatic cerebrovascular injuries and complications. 
